# Biological Responses in the Blood and Organs of Rats to Intraperitoneal Inoculation of Graphene and Graphene Oxide

**DOI:** 10.3390/ma15082898

**Published:** 2022-04-15

**Authors:** Soledad Aguado-Henche, María Lorenza Escudero, María Cristina García-Alonso, Rosa María Lozano-Puerto, Celia Clemente de Arriba

**Affiliations:** 1 Department of Surgery-Anatomy and Social Sciences, University of Alcalá (UAH), Ctra. Mad-Barc Km 33,600, Campus Universitario, 28805 Alcala de Henares, Spain; celia.clemente@uah.es; 2 Department of Surface Engineering, Corrosion and Durability, National Center for Metallurgical Research (Centro Nacional de Investigaciones Metalúrgicas, CENIM-CSIC), Avda. Gregorio del Amo 8, 28040 Madrid, Spain; escudero@cenim.csic.es (M.L.E.); cristina.g.alonso@csic.es (M.C.G.-A.); 3 Cell-Biomaterial Recognition Laboratory, “Center for Biological Research Margarita Salas (CIB-MS-CSIC), Department of Cellular and Molecular Biology”, Ramiro de Maeztu 9, 28040 Madrid, Spain; rlozano@cib.csic.es

**Keywords:** graphene, graphene oxide, rats, blood and organs, toxicity

## Abstract

Background: The discrepancy among the in vivo results found in the literature regarding graphene’s side effects led us to conduct an in vivo study with graphene. Methods: In vivo tests involving intraperitoneal inoculation of graphene and graphene oxide nanosheets in rats were carried out to assess potential changes in the blood and organs after 15 and 30 days. Graphene and graphene oxide nanosheets at a concentration of 4 mg per kilogram were suspended in an aqueous solution of 0.9% NaCl at a 1:1 proportion (graphene or graphene oxide), i.e., 1 mg/mL. Results: Optical microscopy of liver, kidney, spleen, and lung tissues revealed no visible histological changes. However, particle traces were found in the peritoneal cavity. Thirty days after inoculation, blood samples were collected for hematological analysis. The blood analysis showed changes indicating a hepatic inflammatory process. Hematological changes after 30 days consisted of alterations to the red series, including microcytosis or higher mean hemoglobin concentrations. In addition, changes in prothrombin and thromboplastin caused longer coagulation times. Conclusion: This study contributes to further clarifying the possible toxicity of graphene and its potential biomedical applications.

## 1. Introduction

Graphene (G) was first isolated from graphite in 2004 [1]. Graphene oxide (GO) is an oxidized derivative of graphene with a size ranging from 10 nm to 1 mm with numerous hydroxyls, carbonyl, carboxyl, and epoxy groups randomly distributed along the carbon network surface providing unique properties [2,3]. Thus, graphene and its derivatives, such as graphene oxide or reduced graphene oxide (rGO), have drawn attention in numerous technological fields due to their magnetic, thermal, and mechanical properties.

The presence of oxygen in the carbon network opens new possibilities for graphene films to improve specific properties of the surfaces on which they are applied. Oxygen can bind graphene to other substances used in disease treatment. However, the experimental results of in vivo applications to date have been inconclusive.

When interacting with living matter, toxicity is the main issue that must be addressed in order to use GO for medical purposes [4]. Therefore, cytotoxic and genotoxic effects must be considered. Cytotoxic and genotoxic effects seem to be very closely related to the potential ability to cross the cellular membrane. The main cytotoxic damage caused by GO nanosheets in the cell is attributed to the interaction of the functional groups within the cell that leads to an increase in the production of reactive oxygen species (ROS) [5]. Several studies have proven that GO can easily enter cells and that this mechanism is strongly dependent on GO flake size [6,7].

The genotoxicity of GO in human mesenchymal stem cells (hMSCs) has been verified by Akhavan et al. [7]. They correlated the ROS level in hMSCs with cytotoxic and genotoxic results. They also found that toxicity strongly depends on the size of the nanomaterial. De Marzi et al. [8] studied pristine GO genotoxic and cytotoxic effects on different cell lines depending on GO flake size. GO potential genotoxicity has received little attention in nanobiomaterial applications in the literature. Nevertheless, toxicity is the main issue that needs to be addressed and explained [6].

GO safety is unclear; some authors have reported that it is safe [9,10,11,12] while others have stated that it induces granuloma formation or mutagenesis [13]. Consequently, further experimental studies are need [14,15]. Furthermore, the in vivo effects depend on the particle size, dose, concentration, route of administration, animal model used, and experimental design.

The potential toxicity in blood is one of the major concerns when these materials are used either in vivo or in vitro. This study was carried out in vivo, although there are in vitro studies that validate its results [16].

Only scattered and contradictory results about the secondary effects produced by graphene derivatives can be found in the literature [10,14,17,18]. This has promoted further in vivo research in order to clarify their effects.

Graphene’s exceptional properties make it potentially suitable for a wide range of biomedical applications [19,20], such as being used as a solid lubricant on metallic surfaces to reduce friction/wear and corrosion processes in joint prostheses. Bearing this application in mind, the analysis of the biological effect of the graphene derivative releases as a consequence of wear processes on tissues and blood is of high priority. The effect of the migration of nanosheets into the blood due to the irregular friction of joint prostheses is a topic that has not been dealt with in the literature. Additionally, to date, the effects of graphene nanosheets have not been assessed in all major organs. With this in mind, this study evaluates the histological and hematological effects of inoculation with G and GO nanosheets through intraperitoneal injections in rats. Then, the aim of the present study was to analyze organ histopathology, blood cell morphology, and serum biochemistry in rats after different inoculation times to gain knowledge on graphene toxicity in vivo in an effort to shed light on its toxicology.

## 2. Materials and Methods

### 2.1. Graphene Derivative

H-grade graphene nanoplatelets acquired from XG Sciences (Lansing, MI, USA) consisted of short stacks of graphene sheets with a platelet shape. Grade H particles have an average thickness of approximately 15 nm and a typical surface area of 50 to 80 m^2^/g. The mean particle diameter was of 5 µm. The smallest platelet size was chosen since it is known that the smallest ones produce the highest toxicity [21].

A graphene oxide aqueous suspension of 4 mg/mL was acquired from Grupo Antolin Holding (Burgos, Spain).

### 2.2. Animals

The in vivo response was studied in male adult Wistar rats of approximately 250 ± 10 g body weight. The rats were allowed to acclimate for seven days in the facilities of the Animal Experimentation Center of the University of Alcalá de Henares (Madrid) prior to the beginning of the experiments. The protocol was reviewed and approved by Madrid’s Ethics Committee for Regional Clinical Research (CEIC-R) and the animals were treated in accordance with the University Ethics Committee, Spanish regulations (RD 53/2013), and the European Union’s guidelines for the care of animals used for experimental purposes (86/609CEE; recommendation 2007/526/CE).

The rats were housed in polycarbonate cages (*n* = 4/cage) and maintained in a controlled environment with a 12-h-dark/12-h-light cycle at a temperature of 22.2 °C and relative humidity of 50–70%. Pellet and nutritionally balanced food and water were available ad libitum. After three days of regular feeding, animal weight and feed consumption were measured and recorded daily at 9:00 a.m.

### 2.3. Surgical Procedure

The experimental design used is summarized in Table 1.

The rats were randomly divided into three different groups of five animals each: control, G, and GO. All groups underwent intraperitoneal inoculation. The in vivo response was assessed 15 and 30 days after inoculation.

The right iliac fosse was chosen as the injection site to prevent damage to the urinary bladder, cecum, and other abdominal organs.

Group 1 (control) was injected with 1 mL of sterile physiological saline solution (0.9% NaCl) in the right iliac fosse. Group 2 (G) was injected with graphene nanosheets at a concentration of 4 mg per kilogram, suspended through probe sonication into an aqueous solution of 0.9% NaCl at a 1:1 proportion (NaCl:G), i.e., a volume of 1 mL at a concentration of 1 mg/mL. Graphene nanoplatelets were handled according to the Occupational Risk Prevention guidelines [22,23], i.e., in the laboratory under an extraction cabin and using masks to prevent powder inhalation. Group 3 (GO) was injected with 1 mL of graphene oxide solution and 0.9% NaCl at a 1:1 proportion at a concentration of 1 mg/mL. The suspension of graphene derivative and physiological saline solution was vigorously stirred just before use in order to avoid the agglomeration of the nanoplatelets [14,15]. Every aliquot was sterilized under vapor at 134 bar for 5 min.

Group 3 (GO) inoculation following the above methodology was only performed after 30 days, based on the previous results of group 2 (G), in order to reduce the number of animals used in the research.

The rats were anesthetized with isofluorane for extraction of 1.5 mL of intracardiac blood and immediately sacrificed in a CO_2_ cabin 15 and 30 days post-inoculation. Organs such as the liver, lung, spleen, kidney, and peritoneal membrane were recovered from the animals for histological analysis.

Before the sacrifice took place, none of the animals showed physical or behavioral changes. Photographs of every rat’s insides were taken immediately after sacrifice.

### 2.4. Histological Examination

The organs were harvested and immersed in 4% paraformaldehyde. Subsequently, they were embedded in paraffin and cut into 4 µm cross sections with a MICROM trademark rotary microtome. The sections were mounted on glass slides and paraffin-embedded sections were dewaxed with xylene, rehydrated through an ethanol series (absolute, 95%, 90%, 80% and 70%), and washed with water. Hematoxylin and eosin (H & E) staining was performed on paraffin sections of liver, lung, spleen, kidney, and peritoneal membrane.

The analysis of isolated cells and particles of the abdomen cavity was performed by washing with physiological serum and subsequent extension on glass slides that were stained with Giemsa.

Microscopic evaluations were conducted using a Zeiss West Germany bright-field microscope.

### 2.5. Hematological Examination

The blood samples were divided into two parts. The first was stored in BD Vacutainer^®^ blood collection tubes containing K3-EDTA (Beckton & Dickton, Franklin Lakes, NJ, USA) for hematological analysis. The second was kept in 5 mL test tubes in order to prepare the serum samples for biochemical analysis. The serum samples were prepared through centrifugation at 3000× *g* for 15 min. Subsequently, the serum was separated and placed in 1.5 mL Eppendorf tubes in a cold chamber for transport to a laboratory.

The hematological analysis was performed by UNILABS (Madrid, Spain). The hematological parameters were analyzed by a Sysmex Kx-21n hematology autoanalyzer (Sysmex Corporation, Kobe, Japan).

The hematological analysis included red and white cells and biochemical parameters. Whole-blood hematological parameters included hemoglobin (Hgb), hematocrit (Hct), white blood cell (WBC) count, red blood cell (RBC) count, mean corpuscular volume (MCV), mean corpuscular hemoglobin concentration (MCHC), and platelet (PLT) count.

The biochemical parameters measured in the serum samples included creatinine (Crea), aspartate aminotransferase (AST), alanine aminotransferase (ALT), lactate dehydrogenase (LDH), total protein (TP), triglyceride (TG), and cholesterol (CHO).

### 2.6. Data Analysis

STATGRAPHICS^®^ Plus version 5.1 (Statistical Graphics Corp., Warrenton, VA, USA) was used for all statistical analyses. The hematological results are presented as mean ± standard deviation (mean ± SD). Significant differences between groups were examined using one-way analysis of variance (ANOVA). Statistically significant mean differences were assessed using ANOVA, followed by Student’s *t*-test for multiple comparisons. ANOVA with a *p*-value below 0.05 was considered statistically significant.

## 3. Results

### 3.1. Macroscopic Observation

**Figure 1:**
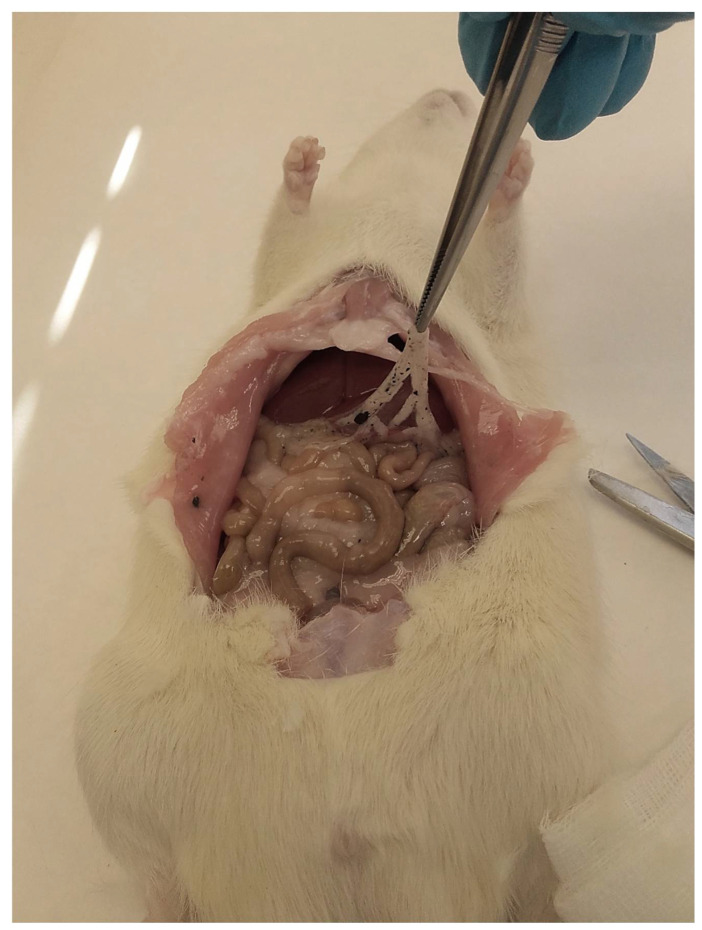
Macroscopic view of graphene deposits in a rat’s abdominal cavity.

The largest aggregates were seen in the proximity of the injection site—in the connective tissue of the abdominal skin, muscles, and peritoneum. Numerous smaller spherical aggregates (around 2 mm diameter) were lodged in the mesentery (Figure 1).

**Figure 2:**
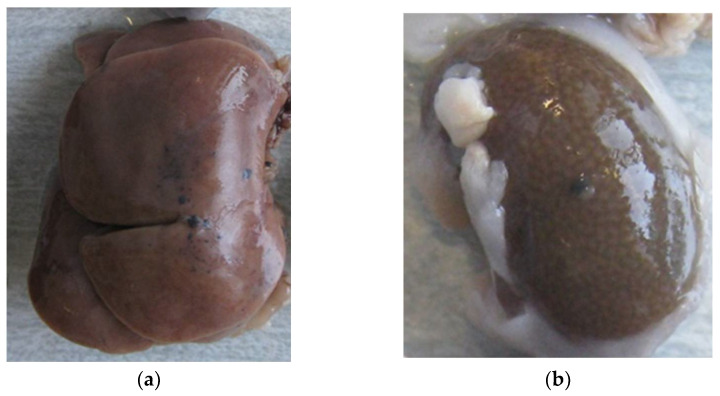
(**a**) Graphene deposits on the liver surface (identified as black dots). (**b**). Graphene deposits on the kidney surface (identified as black dots).

As shown in Figure 2, graphene particles were also found on the liver and kidney surfaces (Figure 2a,b, respectively). In contrast, no deposits were observed on the spleen or lung surfaces.

### 3.2. Histological Examination

No differences in viscera histology were observed between groups. Free particles were detected in the peritoneal fluid samples collected from the abdominal cavity, but with no inflammatory cellular response.

### 3.3. Hematological Results

Group 2, injected with G, presented a slight increase in white blood cell, platelet, and transaminase levels after 15 days (Table 2). Nevertheless, the differences were not significant compared to the control group. The red blood cell and hemoglobin levels were similar in both control and G groups (Table 3).

The small differences detected in the animals after 15 days of treatment led us to extend the study to 30 days, now including a GO group.

Group 2, injected with G, showed an increase in the red blood cell count and reduced hematocrit after 30 days compared with the control group. However, the hemoglobin level was similar to that of the control group. Despite these differences not being significant, the combination of the three factors induced a significant (*p* = 0.0025) decrease in two variables in group 2 (G): red blood cell corpuscular volume and mean hemoglobin concentration per red blood cell, as shown in Table 3. These results indicate microcytosis. This microcytosis has not been found in the scientific literature.

**Figure 3:**
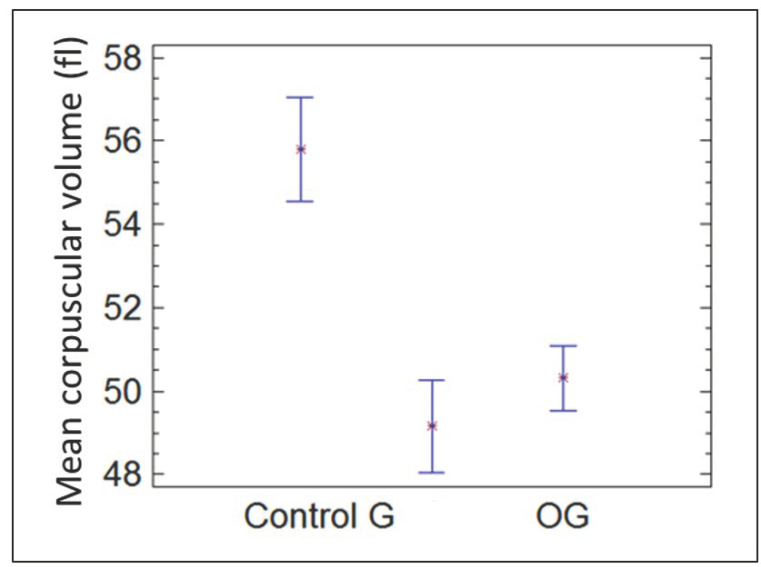
MCV in the control group, group G, and group GO at 30 days.

Figure 3 shows the mean corpuscular volume after 30 days for the groups control, G, and GO. After 30 days, the decrease in red blood cell corpuscular volume remained significant in both groups G and GO.

**Figure 4:**
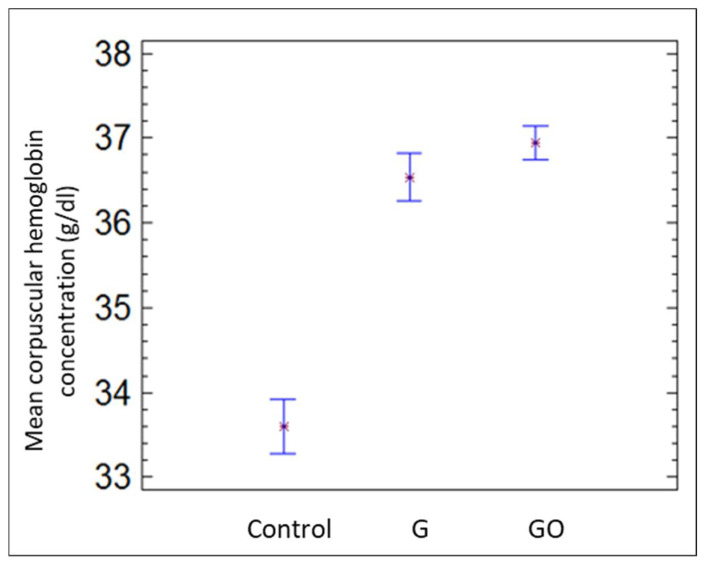
MCHC in the control group, group G, and group GO at 30 days.

**Figure 5:**
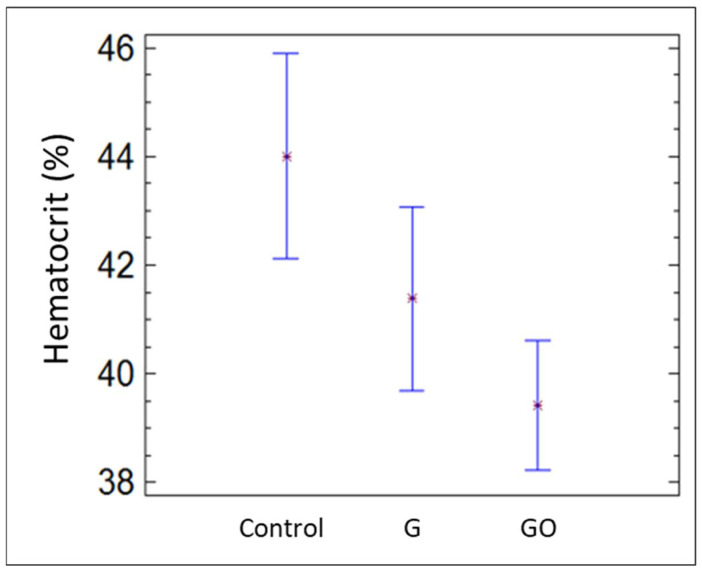
Hct in the control group, group G, and group GO at 30 days.

Figure 4 contains the mean corpuscular hemoglobin concentration for the groups control, G, and GO after 30 days. Mean corpuscular hemoglobin concentration increased significantly in the G and GO groups compared with the control group. Nonetheless, hematocrit decreased in both groups, as shown in Figure 5.

**Figure 6:**
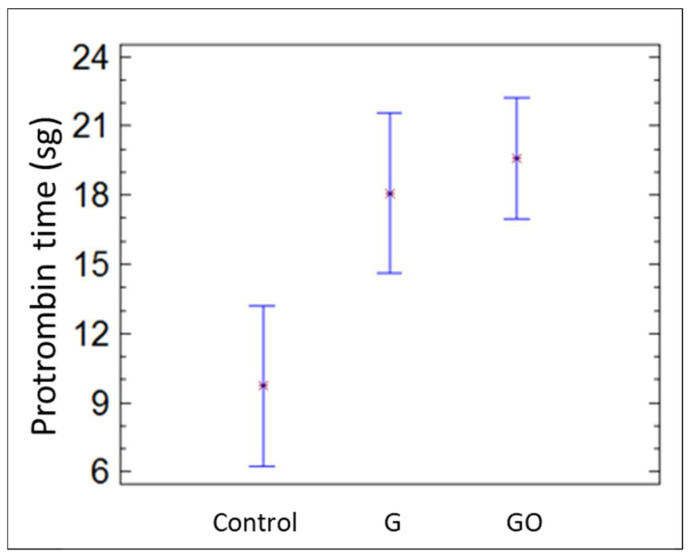
Protrombin time in the control group, group G, and group GO at 30 days.

**Figure 7:**
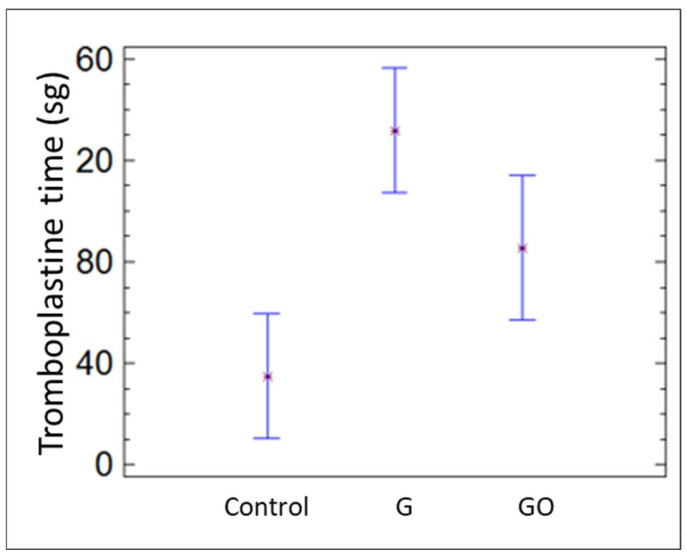
Tromboplastine time in the control group, group G, and group GO at 30 days.

The platelet count was similar in the three groups. Nevertheless, the prothrombin and thromboplastin times were significantly longer in the G and GO groups than in the control group (*p* = 0.037), as shown in Figure 6 and Figure 7, respectively.

Table 2 shows, with regard to the transaminases, that G and GO inoculation produces a significant LDH increase after 30 days compared to the control group. A significant increase in the ALT levels is also observed 30 days after inoculation with G.

Additionally, from Table 2 we can see that plasma proteins, albumin and gamma globulin percentages all significantly increased in the group inoculated with GO after 30 days, while they decreased slightly in the group treated with G, with no significant differences compared to the control group.

Creatinine levels slightly increased in the group treated with G after 15 and 30 days, while the rats inoculated with GO presented values similar to the control group (Table 2).

## 4. Discussion

To date, the results found in the literature about the secondary effects produced by graphene derivatives are contradictory.

In this work, the intraperitoneal injection technique was used prior to carrying out the study using the intraarticular injection technique.

The G inoculation method proved to be easy to apply and well-tolerated by the animals. However, it presented the disadvantage of aggregation in the peritoneal cavity [14,15]. Although G and GO nanosheets were dispersed through ultrasound prior to inoculation, once they were placed in the abdominal cavity the nanosheets aggregated and hardly diffused to adjacent organs. Because of aggregation, these G compounds were handled as solid materials of 2 mm diameter. Therefore, the biological response induced cannot be considered to be due to individual nanosheets but due to a solid mass of about 2 mm.

The effects of G and GO have been studied in more depth with intravenous than with intraperitoneal administration. Nevertheless, the gradual and slower particle release from the peritoneal cavity to the rest of the body may be more similar to the particle release that may occur due to friction of prosthesis joint surfaces, producing chronic toxicity.

Despite GO toxicity having been studied in non-rodent organisms [24], surgical procedures are easier to perform in rats as they lead to fewer errors and greater efficiency in terms of money, time, and animal lives. Additionally, Wistar rats tend to reach a bigger size than Sprague Dawley (SD) or Lewis (LEW) rats [25].

The peritoneal cavity provides a wide surface for drug absorption, which allows for quick transport into the blood flow. Nonetheless, there is a risk of puncturing the small intestine or producing adhesions or severe infections. None of these occurred in our case, and all animals showed a normal response to administration through the intraperitoneal route.

In the current study, the presence of non-absorbed particles in the abdominal cavity indicated that homogeneous blood values were not reached in all animals, as would be the case with particles derived from joint prosthesis. Consequently, this technique seems to be valid for a preliminary study on G and GO secondary effects, as is proven by the results we obtained. The intraperitoneal technique is, however, not valid for studying the secondary effects of different GO concentrations in the acute phase. In this case, direct intravenous inoculation would be the most useful technique. Some authors have recently applied the intraperitoneal technique with increasing GO concentrations, observing dose-dependent toxicity [26]. We consider that the uneven presence of the graphene deposits causes an uneven release into the bloodstream regardless of the dose.

When GO was intravenously administered to mice, it mostly accumulated in the liver and lungs. The GO uptake in the lungs increased with an increasing dose. For medical applications, small-sized GO is recommended [21]. In our case, the absence of lung injury can be explained due to the route of administration chosen. Nevertheless, other authors [27] who used the inhalation route to administer graphene oxide to male Sprague Dawley rats only detected minimal toxic responses in the animals. The histopathological examination did not reveal any changes at low concentrations (1.0 L/min).

Another indicator of liver injury was the increase in the percentages of albumin and gamma globulin at 30 days with GO. As Table 2 shows, there is a non-significant increase in AST; however, the significant increase in LDH at 30 days with G and GO could indicate liver injury at the clinical level.

The increase in albumin percentage could be explained by the rats’ hemoconcentration state. Various authors [10,12,26] agree that G and GO induce liver injury. The present study confirmed it in both cases. Although some authors reported that its toxicity diminished with time [17], such a reduction was not observed in the G group of the present study after 30 days. In a recent study [26], a significant increase in ALT was observed in Wistar rats after intraperitoneal GO administration at a concentration of 10 mg/kg (high dose; more than twice the concentration used in the present study). On the other hand, small-sized GO, like the ones used in this study, with proper functionalization is recommended for medical applications by some authors [21]. However, it has been proven that they may produce greater liver toxicity [13]. The present study shows that it was deposited on the liver surface. A study in mice also proved that GO was quickly cleared from blood and mainly accumulated in the liver and lungs. Large-sized GO (1–5 µm) was accumulated in the lungs, while small-sized GO (110–500 nm) was accumulated in the liver [21]. No lung injury was detected in the present study, which can be explained due to the GO administration technique applied (intravenous vs. intraperitoneal).

Intraperitoneal GO administration produced fewer negative effects on creatinine levels and renal function than G administration (Table 2). Rats inoculated with G and GO presented smaller red blood cells with higher hemoglobin contents. Although these data revealed smaller-size red blood cells (microcytosis), the higher hemoglobin concentration compensates for the smaller cell size. These results agree with the characteristic alterations in the red blood cells described in the literature. Kanakia et al. [28] used high doses (between 25 and 100 mg/kg weight) to analyze toxicity, finding no significant decrease in mass cell volume (MCV). Nevertheless, this feature was not verified by this study, which used a similar dose. Guo et al. [29] suggest that the different in vivo responses found by various authors may be due to the state of the functional groups on the particulate surface and oxygen content/surface charges. Maybe the binding of Fe to the graphene network, especially in the case of graphene oxide (group 3), impairs the transport of oxygen in the cells. This may be explained by the binding of graphene to the hemoglobin iron, which may hinder oxygen transport to cells. This difficulty could be compensated for with a larger number of hemoglobin molecules, but it would not explain the microcytosis, which could be caused by membrane damage [30].

Some in vitro studies claim that G does not induce any complement or platelet activation, meaning that it is compatible with blood [18]. GO can trigger the complement activation and showed significant adverse effects on the activated partial thromboplastin time but not on the prothrombin time in a past study [31]. In the present study, thromboplastin and prothrombin times increased significantly, therefore altering both the intrinsic and extrinsic coagulation pathways. The longer coagulation times, verified by the changes in both prothrombin and thromboplastin, open potential applications for coronary stents, where the use of anticoagulant therapy is needed [32]. According to other authors, intravenous G administration induced strong platelet aggregation and extensive thromboembolism in mice, while GO was less effective at aggregating platelets [33,34]. Although we found coagulation alterations (prothrombin and thromboplastin), we did not observe platelet aggregation effects.

Coagulation result collection is a sensitive process, and incorrect data may be obtained due to mishandling during transport to the laboratory. Regardless, some coagulation mechanisms seemed to be altered, which is in keeping with our results.

## 5. Conclusions

Intraperitoneal G and GO inoculation led to delayed deposits in the peritoneum, liver (causing livery injury), and kidney. The lungs were not affected. Both materials caused an increase in the platelet count (larger with G) and a non-significant decrease in fibrinogen. Prothrombin and thromboplastin times increased both with G and GO, inducing changes in coagulation. Red blood cell corpuscular volume decreased with both G and GO (microcytosis).

This study aimed to contribute to examining graphene’s biomedical applications. However, further studies are needed in order to shed light on its toxicity and potential effects at the histological level.

## Figures and Tables

**Table 1:** Experimental design of in vivo test.

	Animal Groups		
Days	Group 1 (Control)	Group 2 (G)	Group 3 (GO)
15	5	5	-
30	5	5	5

**Table 2:** Effects of G and GO on coagulation and biochemical parameters (30 days).

Parameters	Control (Group 1)	15 Days with G (Group 2)	30 Days with G (Group 2)	30 Days with GO (Group 3)
Leukocytes (10^3^/mm^3^)	4.668 ± 1.22	6.75 ± 2.18	5.72 ± 0.81	3.64 ± 1.43
Basophils (%)	0.30 ± 0.08	0.42 ± 0.13	0.14 ± 0.09	0.07 ± 0.067
Basophils (10^3^/mm^3^)	0.02 ± 0.006	0.03 ± 0.017	0.01 ± 0.015	0.004 ± 0.005
Platelets (10^3^/µL)	660.50 ± 213.48	861.20 ± 305.63	464.00 ± 271.70	668.20 ± 281.62
Protrombin time (s)	9.725 ± 0.33	9.88 ± 0.31	18.08 ± 2.22 *	19.62 ± 6.39 *
Tromboplastin time (s)	34.975 ± 4.55	32.68 ± 5.14	131.75 ± 34.27 ***	85.53 ± 35.38 **
Fibrinogen (mg/dL)	201.90 ± 6.20	191.10 ± 13.15	175.78 ± 113.85	120.01 ± 52.49
Creatinine (mg/dL)	0.30 ± 0.04	0.36 ± 0.09	1.04 ± 0.26	0.28 ± 0.06
Total Proteins (g/dL)	6.40 ± 0.53	6.60 ± 0.10	6.48 ± 0.44	6.26 ± 1.46
AST (U/L)	89.75 ± 18.25	91.20 ± 8.76	99.20 ± 31.12	118.11 ± 122.41
ALT (U/L)	30.30 ± 2.99	34.60 ± 4.22	35.80 ± 6.14 **	18.45 ± 5.42
LDH (U/L)	402.00 ± 98.11	537.80 ± 77.93	3883.50 ± 1471.50 ***	777.67 ± 552.59 **
Total Cholesterol (mg/dL)	90.50 ± 16.22	97.00 ± 4.00	94.00 ± 28.01	84.82 ± 18.94
Triglycerides (mg/dL)	186.00 ± 68.59	271.60 ± 28.08	179.00 ± 85.42	134.58 ± 65.18 *
Beta globulins (%)	18.40 ± 2.24	20.28 ± 0.67	22.68 ± 8.24	15.03 ± 3.50
Albumin (%)	50.10 ± 1.84	30.29 ± 0.16	41.88 ± 12.93	55.41 ± 1.23 *
Gamma Globulin (%)	0.975 ± 0.013	0.86 ± 0.23	0.55 ± 0.25	1.58 ± 0.36 **

Notes: Each value represents the mean ± SD. * *p* ≤ 0.05, ** *p* ≤ 0.01, and *** *p* ≤ 0.001 when compared with control. Abbreviations: AST: aspartate aminotransferase; ALT: alanine aminotransferase; LDH: Lactate dehydrogenase.

**Table 3:** Effects of G and GO on red blood series (30 days).

Groups	RBC (10^6^/µL)	Hgb (g/dL)	Hct (%)	MCV (fL)	MCHC (g/dL)
Control	7.88 ± 0.30	14.75 ± 0.53	44.00 ± 2.04	55.80 ± 0.35	33.60 ± 0.41
G	8.43 ± 0.38	14.82 ± 0.50	41.38 ± 1.15	49.16 ± 2.54 ***	36.54 ± 0.26 ***
GO	7.84 ± 2.01	14.41 ± 3.59	39.43 ± 2.87 *	50.31 ± 1.09 ***	36.94 ± 0.44 ***

Notes: Each value represents the mean ± SD. * *p* ≤ 0.05 and *** *p* ≤ 0.001 when compared with control. Abbreviations: G: graphene; GO: graphene oxide; RBC: red blood cells; Hgb: hemoglobin; Hct: hematocrit; MCV: mean corpuscular volume; MCHC: mean corpuscular hemoglobin concentration.

## Data Availability

Not applicable.
